# Tzfp Represses the Androgen Receptor in Mouse Testis

**DOI:** 10.1371/journal.pone.0062314

**Published:** 2013-04-25

**Authors:** Kari Furu, Arne Klungland

**Affiliations:** 1 Centre for Molecular Biology and Neuroscience, Department of Microbiology, Oslo University Hospital, Oslo, Norway; 2 Institute of Basic Medical Sciences, University of Oslo, Oslo, Norway; Université Paris-Diderot, France

## Abstract

The testis zinc finger protein (Tzfp), also known as Repressor of GATA, belongs to the BTB/POZ zinc finger family of transcription factors and is thought to play a role in spermatogenesis due to its remarkably high expression in testis. Despite many attempts to find the *in vivo* role of the protein, the molecular function is still largely unknown. Here, we address this issue using a novel mouse model with a disrupted *Tzfp* gene. Homozygous *Tzfp* null mice are born at reduced frequency but appear viable and fertile. Sertoli cells in testes lacking Tzfp display an increase in Androgen Receptor (AR) signaling, and several genes in the testis, including *Gata1*, *Aie1* and *Fanc,* show increased expression. Our results indicate that Tzfp function as a transcriptional regulator and that loss of the protein leads to alterations in AR signaling and reduced number of apoptotic cells in the testicular tubules.

## Introduction

The Testis Zinc Finger Protein (Tzfp), also known as Repressor of GATA, is a transcription factor belonging to the BTB/POZ-ZF (Broad complex, Tramtrack, Brick à brack (BTB) or poxvirus and zinc finger (POZ)-zinc finger) protein family [1], [2]. The BTB domain is a widely distributed protein-protein interaction motif that is often found at the N-terminus of zinc finger transcription factors, whereas the C terminal Krüppel C_2_H_2_ zinc fingers are thought to be an important DNA-binding domain. The BTB/POZ-ZF proteins generally function as transcription regulators and are involved in a broad range of biological processes, including cell survival and differentiation [3], [4]. Tzfp is homologous to another BTB/POZ-ZF protein, Plzf (promyelocytic leukemia zinc finger protein). BLAST analysis reveals over 50% identity, with the highest similarity in the zinc finger domain. PLZF is regarded as a tumor suppressor and works as a transcriptional repressor and chromatin remodeller implicated in cancer and the self-renewal of spermatogonial stem cells [5], [6].

Mice deficient of Tzfp have previously been generated [7], [8], revealing that loss of the protein is not embryonic lethal and that mice lacking Tzfp develop without any obvious phenotype. In blood, TZFP is expressed at high levels in the early stages of differentiation but declines during subsequent differentiation into erythroid and myeloid lineages [9]. Disruption of the *Tzfp* gene leads to increased T lymphocyte proliferation, cytokine production and altered hematopoetic stem cell homeostasis [8], whereas overexpression of the protein is accompanied by accumulation in G1 and increased rates of apoptosis. Tzfp thus seems to be a negative regulator of T lymphocyte activation [7]. Recent findings also suggest a role in B lymphocyte differentiation [10]. TZFP makes a heterodimer with the FA group C protein (FANCC) [11], which is one of at least 13 distinct complementation proteins involved in the autosomal recessive disorder Fanconi anemia (FA) [12]. Tzfp also binds GATA-3, one of six members in the GATA transcription factor family [13]. GATA-3 is involved in the development of the T cell lineage and TZFP represses GATA-3-induced transactivation.

**Figure 1:**
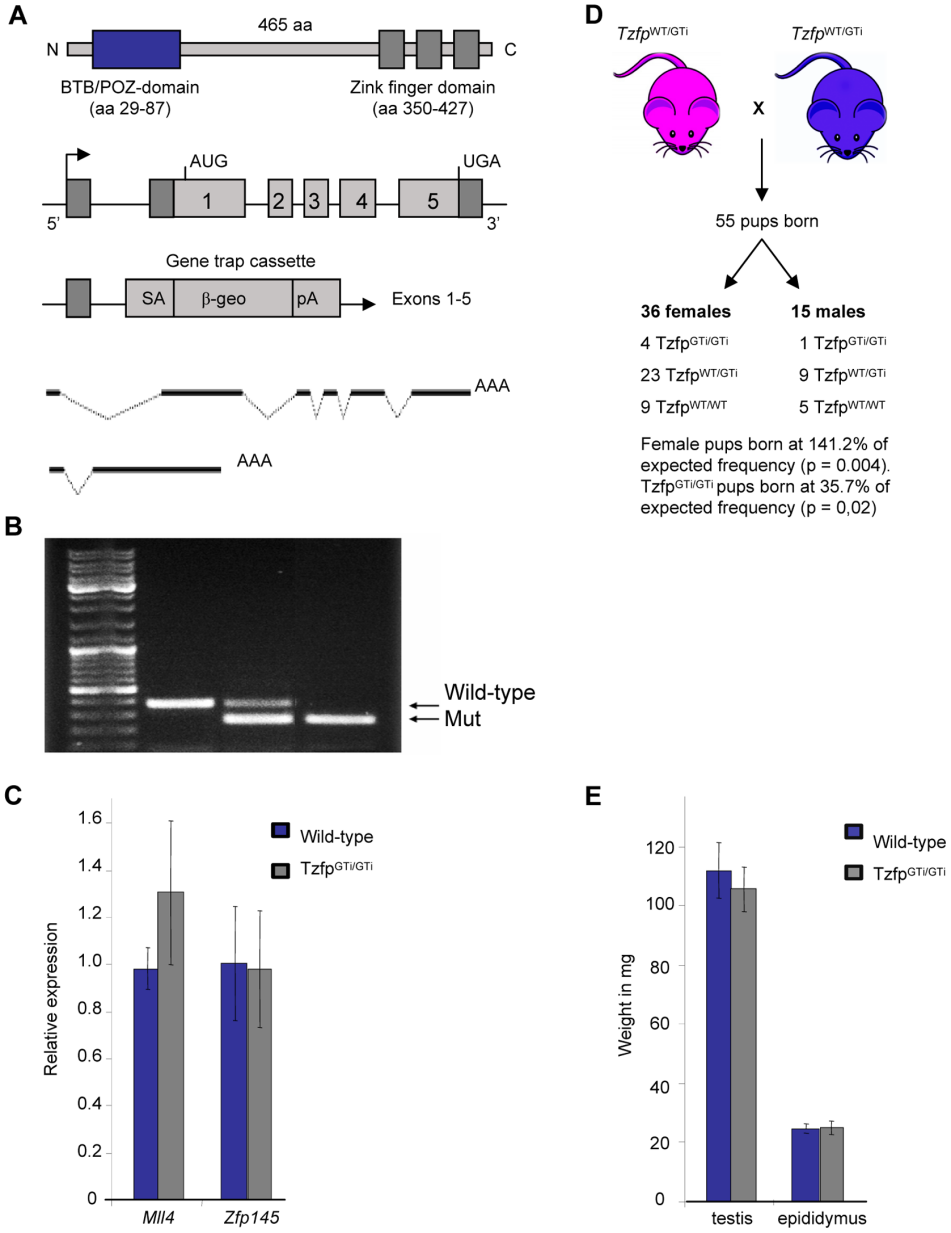
The *Tzfp^GTi/GTi^* mouse model. (A) Overview of the Tzfp protein with the BTB/POZ and zink finger domains (top), and of the wild-type and mutated *Tzfp* allele upon insertional mutagenesis (middle). The wild-type and mutated *Tzfp* transcript is also included (bottom). (B) Genotyping gel showing fragments indicating samples from wild-type, heterozygous and *Tzfp^GTi/GTi^* mice. (C) Gene expression analysis of *Mll4* and *Plzf* in wild-type and *Tzfp^GTi/GTi^* testis reveals no significant difference between wild-type and *Tzfp^GTi/GTi^* testis. (D) Crosses between heterozygous animals reveal a sex-ratio distortion and fewer than expected homozygous mutant pups. The χ2 -test was used to determine significance. (E) Weight distribution of testis and epididymus in wild-type and *Tzfp^GTi/GTi^* mouse reveal a small, but not significant reduction in testis weight in the *Tzfp^GTi/GTi^* mice.

Tzfp probably exists in at least two isoforms [13], [1], [14]. Isoform 1 consists of 465 amino acids (Fig. 1A) and is predicted to be testis specific, whereas the shorter isoform 2 is expressed in B-and T lymphoid cells and lacks the N-terminal BTB/POZ domain [1], [13]. In 2001, Tang *et al.* showed that the zinc finger domain binds to a specific genetic sequence, the Tzfp binding site (tbs) TGTACAGTGT, in the upstream flanking sequence of the *Aie1* gene [15]. They also showed that the BTB domain has a repressive effect, indicating that Tzfp negatively regulates the expression of genes carrying the tbs sequence. *Aie1* encodes Aurora-C, a member of the Aurora kinase protein family, and is primarily expressed in germ cells where it is thought to regulate chromosome segregation and cytokinesis during male meiosis [16].

A link between TZFP and the Androgen Receptor (AR) was recently discovered by Kaufmann *et al.* who show that TZFP can form a trimeric complex with AR and the endogenous retrovirus encoded protein Rec [17]. AR is a member of the nuclear receptor superfamily and functions as a ligand-dependent transcription factor, regulating the expression of an array of androgen-responsive genes [18], [19]. AR and androgens are vital for spermatogenesis [20], and can be detected in Sertoli cells (SC), the Peritubular myoid (PM) cells, and cells in the interstitial spaces including Leydig cells and perivascular smooth muscle cells in the testis [21], [22].

Based on the results of previous studies, Tzfp seem to play a role during the proliferative stages of primitive hematopoetic progenitors and lymphocytes [7], [8], [10], [13], [9], but its precise roles in other forms of cell differentiation remain unknown. The protein is, however, thought to play a role in spermatogenesis due to its high expression in testis [1], [11], [13]. We previously identified Tzfp to interact with the dioxygenase Alkbh1, a histone H2A dioxygenase, and that these two proteins regulate the expression of a piRNA cluster located on chromosome 7 in the mouse genome [23], [24]. A remarkable >1000-fold upregulation of individual piRNAs derived from this cluster was found in pachytene spermatocytes isolated from mouse *Alkbh1*- and *Tzfp*-deficient testes. In the present study we use Tzfp deficient mice to further investigate the role of this protein in spermatogenesis. In agreement with previous studies, we find a remarkably high expression of *Tzfp* in testis [1], [11], [13]. We further show that *Tzfp* is most highly expressed in pachytene spermatocytes during prophase I of meiosis. Mice targeted at both *Tzfp* alleles are born at reduced frequency but appear viable and fertile. Moreover, we show that removal of Tzfp leads to altered regulation of *Fancc, Aie1*, and *Gata-1* in testis, and that it leads to increased Androgen Receptor signaling in Sertoli cells. These results show that Tzfp is a potent repressor of several genes in murine testis and that it is part of a complex system that ensures proper regulation of meiosis.

## Materials and Methods

### Generation of the *Tzfp^GTi/GTi^* Mouse

Embryonic stem (ES) cells containing a gene trap cassette inserted upstream of the *Tzfp* exon 1 were obtained from the Texas Institute of Genomic Medicine (C57Bl/6 gene trap ES cell clone IST12443F7). Briefly, the gene trap vector includes a promoter-less reporter gene (β-GEO) downstream of a splice acceptor (SA) sequence followed by a polyA tail. Insertion of the gene trap cassette terminates transcription prematurely, resulting in a transcript containing none of the exons within the *Tzfp* gene. We injected the IST12443F7 ES cells into C57Bl/6 blastocysts and obtained male mice capable of transmitting the mutated *Tzfp* allele.

The gene trap insertion site was determined using iPCR-based direct-sequencing protocol. ES cells were microinjected into blastocysts and implanted into recipient female mice to permit development of the embryos into chimeras, and subsequent breeding was performed to select founders with germ line transmission of the gene trap allele. The mice were maintained on a C57Bl/6 background.

All mouse experiments were approved by the Norwegian Animal Research Authority (Ref. nr. 08/109551) and done in accordance with institutional guidelines at the Centre for Comparative Medicine at Oslo University Hospital. Animal work was conducted in accordance with the rules and regulations of the Federation of European Laboratory Animal Science Association’s (FELASA). Mice were weaned between 3 and 4 weeks of age, and genotyped by PCR on genomic DNA prepared from ear clips. Genomic DNA was prepared by incubation in 75 µL Hot Shot Lysis Buffer (25 mM NaOH, 0.2 mM Na_2_EDTA, pH 12) at 95°C for 30 min, and then cooled down to 4°C before adding 75 µL Hot Shot Neutralization Buffer (40 mM Tris-HCl, pH 5). Samples were PCR amplified for 40 cycles with an annealing temperature of 58°C using the following primers:

Wild-type allele Tzfp gene:

*5′-ACTGTGGCAGACTAATACTT-3′.*

*5′-GCTCAACAAGTCAAGACTTT-3′.*

*Targeted allele Tzfp gene:*

*5′-ACTGTGGCAGACTAATACTT-3′.*

*5′-CTTGCAAAATGGCGTTACTTAAGC-3′.*

### StaPut Isolation of Pachytene Cells

Pachytene spermatocytes and round spermatids were isolated from 12-week old testes using an adapted version of the StaPut method [25]. Six 12-week old male pups were sacrificed using CO_2_ and the testes were detunicated and the tubules treated with DNase1 (200 µg) and collagenase (10 mg) in 10 ml DMEM for 8 min at 34°C. The tubules were then incubated with DNase1 (200 µg), collagenase (10 mg), trypsin (10 mg) and hyaluronidase (15 mg) in 10 ml DMEM for 15 min at 34°C (all enzymes from Sigma-Aldrich). These steps remove connective tissue and extratubular cells, yielding a cell suspension of germinal cells in DMEM containing 0.5% BSA. The cell suspension was loaded into the cell loading chamber of the StaPut apparatus and subsequently separated by sedimentation velocity at unit gravity in a 2–4% w/v BSA gradient in DMEM medium at 4°C for 2.5 hours. After sedimentation, 10 ml fractions were collected, and the fractions containing pachytene spermatocytes and round spermatids were found based on size and morphological features. Pure fractions were pooled and the cell number counted on a Countess® Automated Cell Counter (Invitrogen). The cells were spun down and the cell pellet was snap frozen in liquid nitrogen. An aliquot of purified cells was fixed on glass slides using Cell Adherence Solution (Crystalgen) on SuperFrost Plus slides (VWR) for microscopic analyses. One separation yielded approximately 1.5×10^6^ pachytene cells with an average diameter of 12.5 µm and 1×10^7^ round spermatids with an average diameter of 9.9 µm.

### TaqMan® Gene Expression Analysis

Total RNA was isolated from cells and tissue using the Fast RNA Pro Green Kit (MP Biomedicals) or the mirVana™ miRNA Isolation Kit (Ambion) according to the manufacturers’ protocol. Any DNA remnants were removed using TURBO DNase (Ambion), and cDNA syntesis was made using High Capacity cDNA Reverse Transcription Kit (Applied Biosystems). The RT-PCR reactions were carried out on a StepOnePlus instrument using 50 ng cDNA, TaqMan® Fast Universal PCR Master Mix and appropriate TaqMan primers and probes (all from Applied Biosystems). GAPDH was used as endogenous control in all assays except in the mouse tissue analysis where 18 s was used. The following pre-designed TaqMan® probes were used:

*GAPDH:* Mm99999915_g1.

*18s:* Hs99999901_s1.

*Tzfp:* Mm00491292_g1.

*Fancc:* Mm01301576_m1.

*AuroraC:* Mm03039428_g1.

*Mll4:* Mm01175395_g1.

*Plzf:* Mm01176865_m1.

*Gata-1:* Mm01352636_m1.

*Gata-4:* Mm00484689_m1.

Tissue and cells from 12 week old animals were used unless stated otherwise. The expression profiling was done using the relative quantitative method ΔΔC_T_. All samples were run in triplicates and with two technical parallels (2 runs per sample). A minimum of two biological parallels were used for each organ, and testis from 6 animals were pooled when performing analysis on germ cells. Statistical analysis was performed using non-paired, two-tailed Student’s *t* test.

### *In Situ* Hybridzation

Testes from 12 week old mice were removed and subsequently placed in Tissue-tek O.C.T Compound (Sakura) on dry ice. The samples were cut into 15 µm thick slices onto SuperFrost Plus slides (VWR) and allowed to air dry. Sections were labeled with DIG-RNA sense and anti-sense probes, covering an area of 801 bases (base 238 to 1038) of the *Tzfp* transcript. The probes were made using a PCR product with T7 and T3 sequence flanking the two strands, and DIG RNA labelling mix (Roche). The following primers were used:

Forward primer with T3 sequence: 5′-**aattaaccctcactaaagg**ggagagagtatagagctaca-3′.

Reverse primer with T7 sequence: 5′-**taatacgactcactatagg**gcgagcaggagagggtaaag-3′.

*In Situ* hybridization was performed using an adapted version of the protocol by Hoover and Goldman [26]. The sections were fixed in 4% paraformaldehyde, dehydrated in 100-50% ethanol and rinsed in 2×SSC (150 mM NaCl and 15 mM sodium citrate, pH 7.4). Permeabilization was performed using 10 µg/ml Proteinase K (Roche) in 0.1 M Tris–HCl, pH 7.5 with 50 mM EDTA for 15 min at 37°C. Sections were post-fixed in 4% paraformaldehyde and treated with 0.2 M HCl for before acetylation in 0.1 M triethanolamine (TEA), pH 7.5, with 0.25% acetic anhydride. Sections were dehydrated and air dried before incubation with hybridization solution (10 mM Tris–HCl, pH 7.5, 50% formamide, 0.3 M NaCl, 1 mM EDTA, 10% dextran sulfate (Fluka) and 1% blocking solution (Roche)), containing 100 ng DIG-RNA probe. Slides were incubated at 55°C over night and then rinsed. Unhybridized RNA was digested by incubation with RNase (50 µg/ml) in NTE buffer (10 mM Tris pH 7.5, 0.5 M NaCl, 1 mM EDTA) for 30 min at 37°C. Slides were then washed in the NTE buffer for 30 min at 60°C. Unspecific staining was blocked by incubating in 2×SSC with 0.05% Triton X-100 and 1% blocking solution for 2 hours before incubation with anti-DIG-alkaline phosphate (1∶1000) (Roche) in MAB buffer (100 mM maleic acid, 150 mM NaCl, pH 7.5) over night at 4°C. The sections were washed in MAB and incubated with equilibration buffer (100 mM Tris–HCl, 100 mM NaCl and 50 mM MgCl) before detection with NBT (Nitro blue tetrazolium chloride) and BCIP (5-bromo-4-chloro-3-indolyl phosphate, toluidine salt) tablets (Roche), and counterstained with haematoxylin. Pictures were taken using an AxioCam ICc1 camera on an Axio Observer.Z1 microscope (Carl Zeiss).

### Immunofluorescent Staining of Frozen Testis Sections

Ten µm thick frozen sections were cut onto SuperFrost Plus slides (VWR) and allowed to air dry. Before staining, the sections were fixed in 4% Pfa for 10 minutes, permeabilized in PBS with 0.5% Triton for 5 minutes and blocked in PBS containing 5% BSA and 5% normal Goat Serum (Invitrogen) for 30 min at room temperature. The slides were then incubated with primary antibodies overnight at 4°C or 37°C prior to detection with secondary antibodies. Primary antibodies used were rabbit anti-AR (1∶50, Abcam), rabbit anti-GATA-1 (1∶100, Cell Signaling), chicken anti-laminin (1∶50, Abcam). Secondary antibodies used were donkey anti-rat Alexa Fluor 594 (1∶500, Invitrogen), and goat anti-rabbit Alexa 594 (1∶500, Invitrogen). The slides were counterstained with DAPI (Invitrogen) and mounted with Mowiol (Merck Biosciences Ltd). Pictures were taken using an AxioCam MR Rev3 camera on an Axio Observer.Z1 microscope with Apotome (Carl Zeiss). The images were processed using AxioVision 4.8 software (Carl Zeiss) and Image J.

### Signal Intensity Measurements

Signal intensity upon immunohistochemistry was measured in selected cells using the ImageJ software. The same conditions and exposure times were used on all samples when comparing cells on different slides. Cells of interest were defined using the Region of Interest (ROI) applications, and a mean intensity was found measuring several cells from three different tubules from each genotype. Statistical analysis was performed using non-paired, two-tailed Student’s *t* test.

### Haematoxylin and Eosin Staining

Paraffin embedded mouse testes were cut in 4 µm thick slices on a microtome and fixed on SuperFrost Plus slides (VWR). The slides were deparaffinated in ClearRite3 and rehydrated in 100-70% ethanol. Staining was performed by incubation in haematoxylin 7211 for 2 min, Blueing Reagent 7301 for 1 min and eosin 71204E for 1.5 min (all solutions from Richard-Allan Scientific). Slides were dehydrated, washed in ClearRite 3 and mounted using Mounting Medium 4111 (Richard-Allan Scientific). Pictures were taken using an AxioCam ICc1 camera on an Axio Observer.Z1 microscope (Carl Zeiss) and the images were processed using AxioVision 4.8 software (Carl Zeiss) and Image J.

### Apoptosis Detection

Paraffin embedded tissue sections from wild-type and knock-out mouse testis were made and deparaffinated and rehydrated as described above. The sections were quickly rinsed in PBS before Proteinase K treatment (20 µg/ml) for 20 minutes at 37°C. TUNEL assay was performed according to the manufacturer’s protocol using Cell Death detection kit, TMR red (Roche). The sections were counterstained with DAPI and mounted using Mowiol (Merck Biosciences Ltd). Slides from at least two different animals from each genotype were used. Pictures were taken using an AxioCam MR Rev3 camera on a microscope Axio Observer.Z1 with Apotome (Carl Zeiss). Slides from 3 different animals from each genotype were used and >100 tubules from each genotype were evaluated and the number of positive cells determined. The average number of apoptotic cells was then determined. Statistical analysis was performed using non-paired, two-tailed Student’s *t* test.

### Western Blot

Proteins were isolated from wild-type and *Tzfp^GTi/GTi^* testis using RIPA buffer (20 mM Tris-HCl (pH 7.5), 150 mM NaCl, 1 mM EDTA, 0.5% NP-40, 0.1% SDS, 0.5 mM PMSF (AppliChem) and 1× Protease Inhibitor Coctail (Sigma)). The protein concentration was measured using Protein assay dye reagent concentrate (BioRad) and 150 µg protein was loaded onto NuPAGE® 10% Bis-Tris Gel 1.0 mm (Invitrogen). After SDS-PAGE, the proteins were blotted onto a PVDF membrane and the membrane was blocked in PBS with 5% skim milk before incubating with primary antibody over night at 4°C. A HRP-conjugated secondary antibody was used and the proteins detected using Immune Star WesternC kit (BioRad).

Primary antibodies used are: Anti-AR (Abcam, 1∶100), anti-GATA-1 (Cell Signaling, 1∶1000), anti-GATA-4 (Santa Cruz, 1∶100) and anti-β-actin (Abcam, 1∶2000). Secondary antibodies used are: HRP-conjugated anti-rabbit (Abcam, 1∶20,000) and HRP-conjugated anti-goat (Abcam, 1∶20,000).

## Results

### Tzfp Deficient Mice are Viable and Fertile

**Figure 2:**
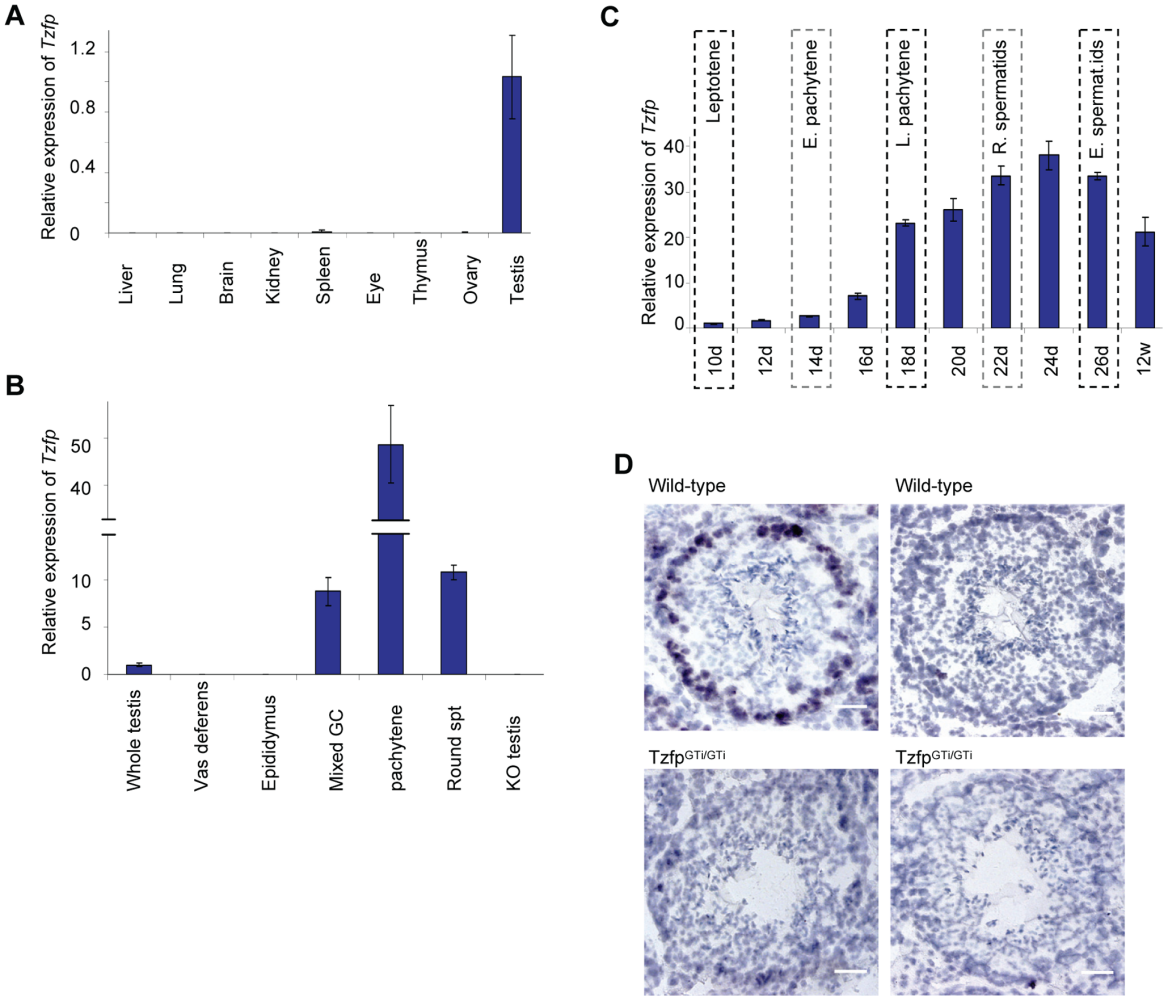
Tzfp gene expression peaks in pachytene spermatocytes. (A) RT-PCR results showing expression of *Tzfp* in various tissues and organs. (B) Expression of *Tzfp* in testis and meiotic germ cells. (C) A diagram of *Tzfp* expression in developing, juvenile and adult testis with the highest developed germ cell present indicated in the top panel. (D) *In Situ* hybridization with DIG labeled *Tzfp* probe on testis cryo sections. Wild-type (top) and *Tzfp^GTi/GTi^* testes (bottom) are shown. Left panel shows tubules hybridized with anti-sense probe, indicating positive pachytene spermatocytes in the wild-type (dark color), and absence of signal in the *Tzfp^GTi/GTi^* sample. Right panel shows tubules hybridized with sense probe, indicating no presence of a sense transcript. The slides are counterstained with haematoxylin (light blue). Scale bar: 20 µm. Abbreviations: GC: germ cells, E./L. pachytene: early and late pachytene spermatocyte, R./E. spermatids: round and elongated spermatid.

A Tzfp mutant mouse was generated using C57BL/6 ES cells containing a splice acceptor site from the gene trap vector (Omnibank Gene Trap Vector 76) upstream of exon 1 of the *Tzfp* gene (FIG 1A). Genotyping confirmed the presence of heterozygous animals in the first litter and later homozygous animals (FIG 1B). RT-PCR analysis showed an average CT value reduction of >9 in the homozygous mutant mouse compared to wild-type (avg. C_T_ value = 22.8 in wild-type and 33.0 in *Tzfp^GTi/GTi^* testes), indicating a decrease in transcript amount of >99%. Absence of the *Tzfp* transcript was also confirmed through *In Situ* hybridization on testis sections (FIG 2D). Mice homozygous for the insertion are hereafter called *Tzfp^GTi/GTi^*.

Another gene located just 1.0 kb upstream of the *Tzfp* gene was identified. This neighboring gene, *Mll4* (mixed-lineage leukemia), is suggested to function as a methyltransferase capable of methylating H3K4 [27]. The expression of *Mll4* was analyzed by RT-PCR to make sure that the expression of this gene was not affected in the *Tzfp^GTi/GTi^* mouse. As described above, Tzfp is homologous to Plzf, a protein encoded by the *Zfp145* gene. The expression of this gene was analyzed to confirm that Plzf does not work as a backup protein in our model, masking any effect of the *Tzfp* deletion. No significant changes in *Mll4* or *Zfp145* expression was observed in the *Tzfp^GTi/GTi^* mice when compared to wild-type (p>0.05) (FIG 1C).

Intercrossing of heterozygous animals obtained normal, fertile homozygous pups with no apparent phenotype. The observation that Tzfp is not essential for the survival of mice pups or the formation of gametes, is in concurrence with the findings of other groups [7], [8]. During the first generations of heterozygous breeding (number of litters = 10), we observed a deviation from the expected frequencies of sex- and genotype distributions (FIG 1D). Transmission ratio distortion (TRD) led to a frequency of homozygous null mice of only 10% rather than the expected 25% (p = 0.02 using χ^2^ test). In addition, the average litter size was 5.1, significantly smaller than the average litter size of 7.0 pups per litter for C57BL/6J mice, indicating loss of homozygous embryos. Breeding revealed a sex ratio distortion yielding 70% female pups rather than the expected 50% (p = 0.003 using χ^2^ test). The male homozygous null mice had testes of size and weight slightly lower than average (p<0.05), but normal epididymus (p>0.5) (FIG 1E).

### The Tzfp Transcript is Most Highly Abundant in Pachytene Spermatocytes in the Testis

We found *Tzfp* to be most highly expressed in testes (FIG 2A). This correlates well with the findings of other groups [1], [11], [13]. In order to identify which cells in the male mouse germline that expresses the *Tzfp* gene, we isolated pachytene spermatocytes and round spermatids from adult animals (12 weeks) using unit gravity sedimentation. RT-PCR analysis showed *Tzfp* expression to be highly elevated in pachytene spermatocytes (FIG 2B), where the gene is upregulated by approximately 50 fold compared to whole testis. The transcript can also be detected in round spermatids, although at a lower level. There is no expression in the vas deferens and epididymus, indicating absence of *Tzfp* in mature spermatozoa.

To further investigate the Tzfp expression, C57BL/6J male pups were sacrificed at specific time points corresponding to the appearance of different spermatocytes and spermatids in the first wave of spermatogenesis in the juvenile mouse [25]. The *Tzfp* expression rapidly increases as spermatogenesis progresses through prophase I (FIG 2C). The *Tzfp* transcript amount increases rapidly from day 16, indicating high expression from mid-pachynema [28], [25]. The expression continues to increase as more cells expressing *Tzfp* develop, and peaks at day 24. To verify the high expression of *Tzfp* in pachytene spermatocytes, we performed *In Situ* hybridization on frozen sections using a DIG-labeled RNA probe (FIG 2D). Most tubules have a clear ring of positive cells, indicating *Tzfp*-expressing pachytene cells. There is no signal in the *Tzfp^GTi/GTi^* samples and in the samples incubated with sense-probe.

### Removal of the Tzfp Protein Leads to Alterations in Testicular Gene Expression

**Figure 3:**
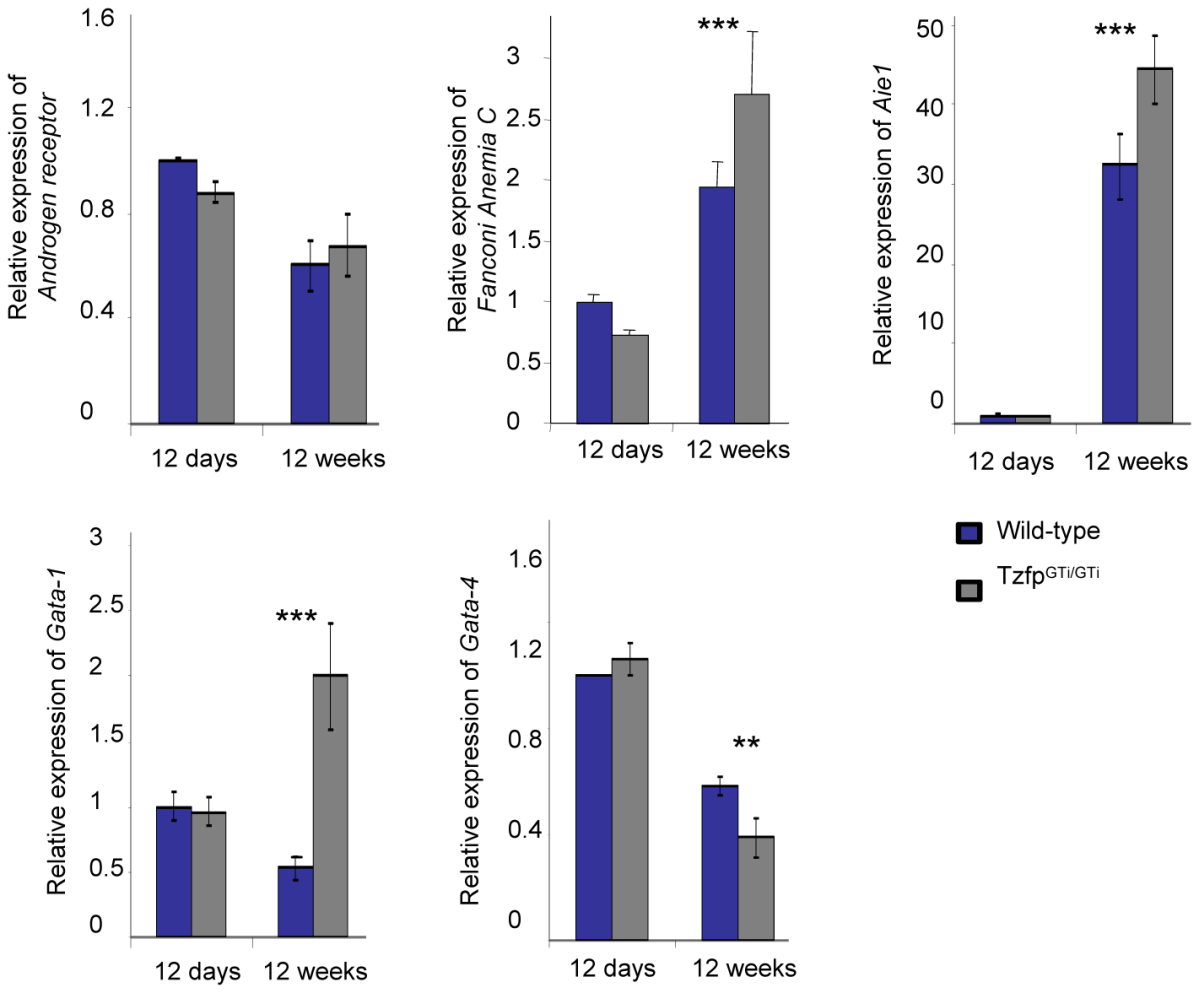
Gene expression analysis. RT-PCR results showing gene expression in wild-type and *Tzfp^GTi/GTi^* testis at age 12 days and 12 weeks. Transcripts analyzed are *Androgen Receptor*, *Fanconi anemiaC*, *Aie1*, *Gata-1* and *Gata-4*. Statistical analysis was performed by using the two-tailed Student’s *t* test: *p<0.05; **p<0.01; ***p<0.001.

In order to identify a possible pathway in which Tzfp is involved, RNA was isolated from juvenile (12 dpp) and adult (12 weeks) testes (i.e. when *Tzfp* expression is low/absent and high, respectively), and the expression pattern of several candidate genes was analyzed. A possible link between Tzfp and the Androgen Receptor has previously been identified [17], yet no significant difference was found in the expression pattern of the AR transcript between wild-type and *Tzfp^GTi/GTi^* testes (FIG 3). TZFP has also been linked to the Fanconi Anemia pathway due to its ability to bind to the FA factor FANCC [11]. Our RT-PCR analysis show that *Fancc* is significantly upregulated in the adult *Tzfp^GTi/GTi^* testis when compared to the wild-type (p<0.001) (FIG 3).

We also wanted to investigate whether loss of *Tzfp* has an effect on the *Aie1* expression. As described above, Tang *et al.* have proposed a mechanism in which Tzfp represses this gene by binding to the tbs motif in the *Aie1* flanking region [15]. In line with these observations, we showed a significant upregulation of *Aie1* expression in *Tzfp^GTi/GTi^* adult testes when compared to wild-type (p<0.001) (FIG 3).

Tzfp has been described as a repressor of GATA [13], and we therefore studied the expression levels of *Gata* mRNAs and proteins in *Tzfp^GTi/GTi^* testis. GATA-1, 4 and 6 are expressed in the Sertoli cells of the testis where they serve to drive spermatogenesis [29], [30], [31]. GATA-1 is expressed in a stage-specific manner, where the strongest signal is found in the Sertoli cells in stage VII-XI seminiferous tubules [32]. RT-PCR analysis revealed that *Gata1* expression is significantly upregulated (p<0.001) in adult *Tzfp^GTi/GTi^* testis when compared to wild-type (FIG 3), indicating a role for Tzfp in the regulation of GATA-1 in testes. A slight downregulation was found in the expression of *Gata-4* in adult testis (p = 0.01) (FIG 3). As expected, no differences in gene expression were observed in juvenile testes where Tzfp is absent (FIG 3).

### Removal of the Tzfp Protein Leads to Increased Amount of Androgen Receptor Signaling in Sertoli Cells

**Figure 4:**
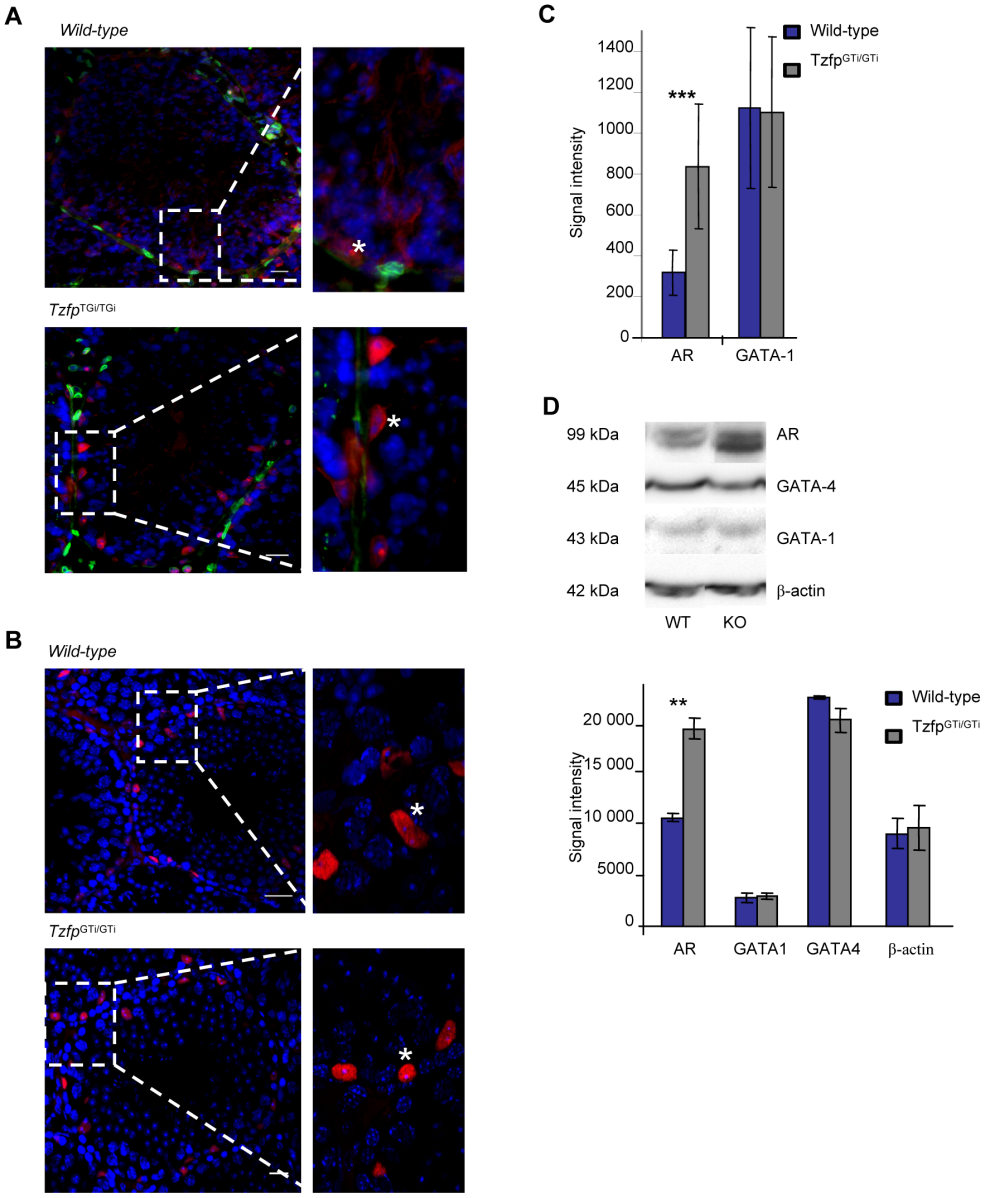
Expression of Androgen Receptor and GATA in wild-type and ***Tzfp^GTi/GTi^*** **testis.** Left panel showing sections of adult wild-type and *Tzfp^GTi/GTi^* testes incubated with anti-AR (A) and anti-GATA-1 (B) (both displayed in red). Anti-AR samples are co-stained with anti-laminin (green). The slides are counterstained with DAPI (blue). Star (*) in magnified area indicates Sertoli cell. Scale bar: 20 µm. C) Quantification of the antibody signal. Statistical analysis was performed using the two-tailed Student’s *t* test: *p<0.05; **p<0.01; ***p<0.001. D) Western blot analysis showing the presence of Androgen Receptor, GATA-1 and GATA-4 in adult wild-type and *Tzfp^GTi/GTi^* testes. β-actin is used as a loading control.

The Androgen Receptor is important for normal progression through spermatogenesis and is expressed by the Sertoli cells and the Peritubular myoid cells (PTMs) within the testicular tubules [33], [34], [35]. As described above, no significant differences in *AR* expression were found between wild-type and *Tzfp^GTi/GTi^* testes (FIG 3), indicating that Tzfp has no effect on *AR* transcription. However, when staining wild-type and *Tzfp^GTi/GTi^* testis sections with anti-AR, a stronger signal in the nuclei of Sertoli cells in the *Tzfp^GTi/GTi^* samples were detected (FIG 4A). This indicates that AR localization, turnover and/or activity are altered upon removal of Tzfp. This, in turn, may indicate a possible role for Tzfp as an AR regulator. As expected, anti-AR revealed similar AR levels in wild-type and *Tzfp* deficient juvenile testes. Quantification of the signal intensity in Sertoli cells from adult testis revealed a significant difference (p = 0.001) between *Tzfp^GTi/GTi^* and wild-type samples (FIG 4C). The data was confirmed by Western analysis, showing AR upregulation in the *Tzfp^GTi/GTi^* samples (FIG 4D). The strongest AR signal was seen in stage VII-VIII tubuli, which contain preleptotene and mid-pachytene spermatocytes, and step 7–8 and 16 spermatids. A strong AR signal in these stages has previously been established [22], indicating a crucial role in AR signaling in these cell types.

Despite the upregulation of *Gata-1* observed in *Tzfp^GTi/GTi^* testes, immunostaining with anti-GATA-1 revealed no difference in signal intensity when comparing *Tzfp^GTi/GTi^* testis sections with wild-type (FIG 4B–C). No significant difference was detected for GATA-1 and GATA-4 with Western analysis (FIG 4D), indicating that lack of Tzfp does not alter the production of these GATA proteins in the testis.

### Tzfp Null Mice Display Normal Progression through Spermatogenesis, but have a Decreased Amount of Tubular Apoptosis

**Figure 5:**
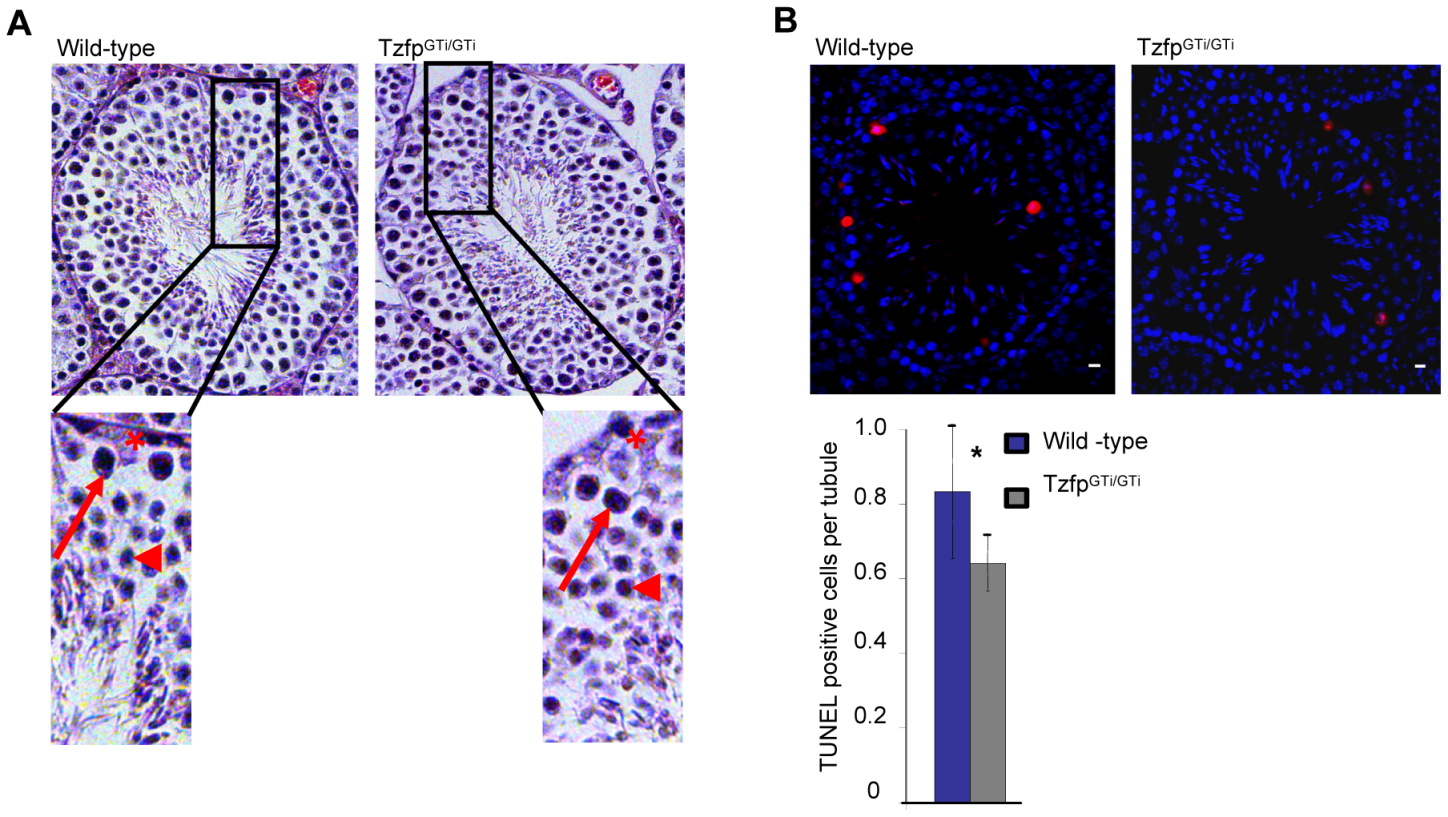
The *Tzfp^GTi/GTi^* testis develops normally but has a reduced number of apoptotic cells. (A) Stage VII-VIII tubules from wild-type (left) and *Tzfp^GTi/GTi^* (right) testis. Magnified areas show the presence of spermatogonia (star), pachytene spermatocyte (arrow) and round spermatid (arrowhead). Scale bar: 20 µm. (B) TUNEL assay performed on wild-type (left) and *Tzfp^GTi/GTi^* (right) testis sections. The histogram indicates quantification of positive cells. Statistical analysis was performed using the two-tailed Student’s *t* test: *p<0.05; **p<0.01; ***p<0.001.

We investigated wild-type and *Tzfp^GTi/GTi^* testis by staining sections with haematoxylin and eosin (HE) to see whether the increased AR signal and changed gene expression in the *Tzfp^GTi/GTi^* mouse testis results in abnormalities in testis anatomy. Stage VII–VIII tubules were of particular interest as these stages contain cells with high *Tzfp* expression, are androgen sensitive, and have high expression of GATA-1. The histological sections, however, looked normal and appear to contain all spermatogenic cell types (FIG 5A). This is not surprising, as the mice are fertile and display normal sexual behavior. The tubular and luminal area was measured and we found that both parameters were slightly smaller in the *Tzfp^GTi/GTi^* testis compared to the wild-type (p = 0.01 and 0.001, respectively) (data not shown). This is in accordance with the slightly smaller testis size found in the *Tzfp^GTi/GTi^* mouse (FIG 1E). We also counted the number of Sertoli cells per tubuli and the Sertoli cell nuclear area in wild-type and adult *Tzfp^GTi/GTi^* testis. No differences were found (data not shown).

We wanted to see whether the *Tzfp^GTi/GTi^* testis had an altered rate of apoptosis in the testis tubules. For this purpose we performed a TUNEL assay followed by statistical testing to see if absence of Tzfp gave increased cell death. Surprisingly, the number of positive cells was lower in *Tzfp^GTi/GTi^* mice compared to in wild-type (p = 0.02) (FIG 5B).

## Discussion

In the present study we show that removal of the Tzfp protein is not lethal, but that breeding mice heterozygous and homozygous for the gene trap insertion yields fewer *Tzfp^GTi/GTi^* pups than expected, indicating decreased survival of *Tzfp* deficient embryos. The protein has previously been shown to be a negative regulator of proliferation and differentiation in hematopoetic cells [7], [8], [10], [13], [9]. Lack of Tzfp leads to increased lymphocyte proliferation and increased cytokine production upon antigen stimulation [8], [7], whereas enforced expression is associated with decreased cytokine production and accumulation in the G_1_ phase of the cell cycle followed later by apoptosis [13], [9]. Tzfp thus seems to play a role in the proliferative stages of hematopoetic cells, but its precise roles in other forms of cell differentiation remain unknown. Although the protein has its highest expression in testis, it is dispensable for the formation of germ cells in both the male and female mouse. Our lab previously identified a role for Tzfp in the PIWI-piRNA pathway [23], but its precise role in spermatogenesis is still unclear. The gene is most highly expressed during pachynema, which is the stage in prophase I of meiosis where the synapsis of homologous chromosomes is completed and crossing over occurs.

Consistent with the presumed role of TZFP as a transcriptional regulator, the protein has been found to localize to distinct nuclear speckles at or near sites of DNA replication [11], [9]. Several BTB/POZ-ZF proteins work as transcriptional regulators through the activation of HDACs via recruitment of the transcriptional co-repressors SMRT and N-CoR [36], [37]. Studies indicate that TZFP work through a similar mechanism, conferring repression of histone acetylation of target cytokine genes in lymphocytes by binding to the genes and recruiting HDAC1 and HDAC2 [38]. Overexpression of TZFP in myeloid cell lines is accompanied by increased rates of apoptosis [9]. We found that Tzfp deficiency leads to a reduced number of apoptotic cells in the testicular tubules from *Tzfp^GTi/GTi^* mice. Interestingly, we found *Fancc*, which encodes the Tzfp interaction partner Fancc, to be upregulated in the *Tzfp^GTi/GTi^* mouse. This protein prevents apoptosis in hematopoietic cells [39], probably through its role in mutational repair of endogenously generated abasic sites [40]. Combined, these results indicate that Tzfp might play a role in cell faith determination during proliferation and differentiation.

*Tzfp^GTi/GTi^* testes have increased Androgen Receptor signaling in the nuclei of Sertoli cells in testicular tubules when compared to wild-type. The signal was found to be increased 3-fold, indicating that Tzfp has a direct or indirect repressive effect on AR. The Sertoli cells (SCs) are somatic cells that nurture and support developing germ cells [41]. They communicate with adjacent cells through specialized cell junctions called gap junctions, ectoplasmic specializations (ES), and tubulobulbar complexes, through which nutrients and locally produced signaling molecules can be transported [42], [43], [35]. SCs extend from the base to the apex of the seminiferous epithelium, interacting directly with developing germ cells throughout spermatogenesis. This allows for the SCs to receive, integrate, and emit signals required for the spermatogenic process, making them indispensable for the development and movement of germ cells [44], [41], [43]. Studies have demonstrated that not only do SCs secrete factors important for the developing germ cells, but the germ cells can also induce transcription and enhance protein synthesis and/or secretion in Sertoli cells [45], [46], [47]. Thus, even though Tzfp and the androgen receptor are not expressed in the same cell types, it is possible that *Tzfp* expression in pachytene spermatocytes affects Sertoli cells, leading to alterations in AR signaling.

Sertoli cells are important for all phases of gametogenesis, including germ cell proliferation, meiosis and differentiation [41]. Studies with a Sertoli cell selective AR knockdown (SCARKO) have revealed that the presence of AR in Sertoli cells is vital for a successful completion of spermatogenesis [44], [48]. In the SCARKO mouse, no germ cells develop to elongated spermatids, rendering mutant male mice infertile. This is probably due to the strong dependence on androgens for mid-pachytene spermatocytes and stage 7 spermatids, which are found in stage VII-VIII tubuli [22], [34]. AR signaling fluctuates with the spermatogenic cycle and peaks in these stages [22].

The GATA factors are other candidates for proteins under Tzfp control that may regulate AR activity. The gonads are prominent sites of GATA expression, with detectable levels of GATA-1, 2, 4 and 6 [49], [50]. Of these factors, only GATA-2 is expressed in the germ-cells [49], whereas Sertoli cells express GATA-1, 4 and 6. As described above, Tzfp is known to be a repressor of GATA, and an interaction between TZFP and the GATA factors 2 and 3 have been shown [13], [51]. However, all vertebrate GATA proteins share a conserved DNA and protein-binding domain composed of two zinc fingers [52]. This leads to an apparent redundancy in binding properties to target sequences, indicating that other GATA members might also bind TZFP.

GATA-1 exerts a temporal expression in Sertoli cells [50], [30] and is only found in stage VI–XII tubules of the seminiferous cycle [31], [32]. This phenomenon appears to be dependent on the presence of maturing germ cells. The expression of GATA-1 coincides with the androgen-sensitive stages of the seminiferous cycle [22] and with the stages containing cells with a high *Tzfp* expression. A 3-fold upregulation of *Gata-1* was apparent in *Tzfp^GTi/GTi^* testis, indicating that Tzfp has a repressive effect on the *Gata-1* gene. This makes GATA-1 perhaps the most likely candidate for a protein under Tzfp control to regulate AR activity. The mechanism through which this occurs is currently unknown, but it is possible that the repression occurs by the recruitment of HDACs. GATA-1 interacts with several HDAC proteins [53], of which HDAC1 has been shown to bind AR and specifically down-regulate AR transcriptional activity [54]. GATA-1 also regulates the androgen receptor corepressor 19 kDa (ARR19), which co-translocates into the nucleus with AR and suppresses AR activity through the recruitment of HDAC4 [55], [56].

Regulation of AR by Tzfp could also occur via Aurora-C, a member of the Aurora kinase protein family. An interaction between Tzfp and the Aurora-C-encoding gene, *Aie1*, has previously been established [15] and in addition, a significant upregulation of this gene was apparent in the *Tzfp^GTi/GTi^* testis when compared to wild-type. The Aurora kinase proteins are serine/threonine kinases essential for cell proliferation and are believed to play important roles in chromosome segregation [57]. Aurora-A interacts with and phosphorylates AR in prostate cancer cells, leading to increased AR activation [58]. Aurora-C has also been associated with cancer formation and transformation, as overexpression of Aurora-C in cell lines induces abnormal cell division resulting in centrosome amplification and multinucleation [59]. Such morphological abnormalities were not observed in the *Tzfp^GTi/GTi^* samples, indicating that the *Aie1* overexpression is not dramatic enough to cause problems with meiotic divisions.

In summary, we generated a viable and fertile *Tzfp^GTi/GTi^* mouse using a gene trap ES clone. We found the gene to be dispensable for life and the formation of germ cells and that the gene is most highly expressed during pachynema, a stage in meiotic prophase I characterized by the occurrence of crossing over. Tzfp most probably has a regulatory role during spermatogenesis and most likely functions as a transcriptional repressor. Removal of the protein leads to a significant increase in the expression of the genes *Fancc, Aurora-C* and *Gata-1,* and an increased AR signaling in Sertoli cells.

## References

[1] Lin W, Lai CH, Tang CJ, Huang CJ, Tang TK (1999) Identification and gene structure of a novel human PLZF-related transcription factor gene, TZFP. Biochem.Biophys.Res.Commun. 264: 789–795. 10.1006/bbrc.1999.1594 10544010

[2] Perez-Torrado R, Yamada D, Defossez PA (2006) Born to bind: the BTB protein-protein interaction domain. Bioessays 28: 1194–1202. 17120193 10.1002/bies.20500

[3] Melnick A, Ahmad KF, Arai S, Polinger A, Ball H, et al (2000) In-depth mutational analysis of the promyelocytic leukemia zinc finger BTB/POZ domain reveals motifs and residues required for biological and transcriptional functions. Mol.Cell Biol. 20: 6550–6567. 10.1128/mcb.20.17.6550-6567.2000 PMC86130 10938130

[4] Kelly KF, Daniel JM (2006) POZ for effect–POZ-ZF transcription factors in cancer and development. Trends Cell Biol. 16: 578–587. 10.1016/j.tcb.2006.09.003 16996269

[5] Barna M, Merghoub T, Costoya JA, Ruggero D, Branford M, et al (2002) Plzf mediates transcriptional repression of HoxD gene expression through chromatin remodeling. Dev.Cell 3: 499–510. 12408802 10.1016/s1534-5807(02)00289-7

[6] Costoya JA, Hobbs RM, Barna M, Cattoretti G, Manova K, et al (2004) Essential role of Plzf in maintenance of spermatogonial stem cells. Nat.Genet. 36: 653–659. 10.1038/ng1367 15156143

[7] Kang BY, Miaw SC, Ho IC (2005) ROG negatively regulates T-cell activation but is dispensable for Th-cell differentiation. Mol.Cell Biol. 25: 554–562. 10.1128/MCB.25.2.554-562.2005 PMC543427 15632058

[8] Piazza F, Costoya JA, Merghoub T, Hobbs RM, Pandolfi PP (2004) Disruption of PLZP in mice leads to increased T-lymphocyte proliferation, cytokine production, and altered hematopoietic stem cell homeostasis. Molecular and Cellular Biology 24: 10456–10469. 15542853 10.1128/MCB.24.23.10456-10469.2004 PMC529048

[9] Dai MS, Chevallier N, Stone S, Heinrich MC, McConnell M, et al (2002) The effects of the Fanconi anemia zinc finger (FAZF) on cell cycle, apoptosis, and proliferation are differentiation stage-specific. J.Biol.Chem. 277: 26327–26334. 10.1074/jbc.M201834200 11986317

[10] Yoon HS, Scharer CD, Majumde P, Davis CW, Butler R, et al.. (2012) ZBTB32 Is an Early Repressor of the CIITA and MHC Class II Gene Expression during B Cell Differentiation to Plasma Cells. J.Immunol. 10.4049/jimmunol.1103371 PMC3424359 22851713

[11] Hoatlin ME, Zhi Y, Ball H, Silvey K, Melnick A, et al (1999) A novel BTB/POZ transcriptional repressor protein interacts with the Fanconi anemia group C protein and PLZF. Blood 94: 3737–3747. 10572087

[12] Moldovan GL, D’Andrea AD (2009) How the fanconi anemia pathway guards the genome. Annu.Rev.Genet. 43: 223–249. 10.1146/annurev-genet-102108-134222 PMC2830711 19686080

[13] Miaw SC, Choi A, Yu E, Kishikawa H, Ho IC (2000) ROG, repressor of GATA, regulates the expression of cytokine genes. Immunity. 12: 323–333. 10.1016/s1074-7613(00)80185-5 10755619

[14] Ikeda R, Yoshida K, Inoue I (2007) Identification of FAZF as a novel BMP2-induced transcription factor during osteoblastic differentiation. J.Cell Biochem. 101: 147–154. 10.1002/jcb.21165 17171645

[15] Tang CJ, Chuang CK, Hu HM, Tang TK (2001) The zinc finger domain of Tzfp binds to the tbs motif located at the upstream flanking region of the Aie1 (aurora-C) kinase gene. J.Biol.Chem. 276: 19631–19639. 10.1074/jbc.M100170200 11279021

[16] Tang CJ, Lin CY, Tang TK (2006) Dynamic localization and functional implications of Aurora-C kinase during male mouse meiosis. Dev.Biol. 290: 398–410. 10.1016/j.ydbio.2005.11.036 16386730

[17] Kaufmann S, Sauter M, Schmitt M, Baumert B, Best B, et al (2010) Human endogenous retrovirus protein Rec interacts with the testicular zinc-finger protein and androgen receptor. J.Gen.Virol. 91: 1494–1502. 10.1099/vir.0.014241-0 20147518

[18] Eder IE, Haag P, Basik M, Mousses S, Bektic J, et al (2003) Gene expression changes following androgen receptor elimination in LNCaP prostate cancer cells. Mol.Carcinog. 37: 181–191. 10.1002/mc.10136 12891627

[19] Jiang F, Wang Z (2003) Identification of androgen-responsive genes in the rat ventral prostate by complementary deoxyribonucleic acid subtraction and microarray. Endocrinology 144: 1257–1265. 12639908 10.1210/en.2002-220718

[20] Maclean JA, Wilkinson MF (2005) Gene regulation in spermatogenesis. Curr.Top.Dev.Biol. 71: 131–197. 10.1016/S0070-2153(05)71005-X 16344105

[21] Kimura N, Mizokami A, Oonuma T, Sasano H, Nagura H (1993) Immunocytochemical localization of androgen receptor with polyclonal antibody in paraffin-embedded human tissues. J.Histochem.Cytochem. 41: 671–678. 10.1177/41.5.8468448 8468448

[22] Bremner WJ, Millar MR, Sharpe RM, Saunders PT (1994) Immunohistochemical localization of androgen receptors in the rat testis: evidence for stage-dependent expression and regulation by androgens. Endocrinology 135: 1227–1234. 8070367 10.1210/endo.135.3.8070367

[23] Nordstrand LM, Furu K, Paulsen J, Rognes T, Klungland A (2012) Alkbh1 and Tzfp repress a non-repeat piRNA cluster in pachytene spermatocytes. Nucleic Acids Res. 10.1093/nar/gks839 PMC3505970 22965116

[24] Ougland R, Lando D, Jonson I, Dahl JA, Moen MN, et al (2012) ALKBH1 is a Histone H2A Dioxygenase Involved in Neural Differentiation. Stem Cells 30: 2672–2682. 22961808 10.1002/stem.1228 PMC3546389

[25] Bellve AR (1993) Purification, culture, and fractionation of spermatogenic cells. Methods Enzymol. 225: 84–113. 10.1016/0076-6879(93)25009-q 8231890

[26] Hoover F, Goldman D (1992) Temporally correlated expression of nAChR genes during development of the mammalian retina. Exp.Eye Res. 54: 561–571. 10.1016/0014-4835(92)90135-f 1623941

[27] Lee S, Lee DK, Dou Y, Lee J, Lee B, et al (2006) Coactivator as a target gene specificity determinant for histone H3 lysine 4 methyltransferases. Proc.Natl.Acad.Sci.U.S.A 103: 15392–15397. 17021013 10.1073/pnas.0607313103 PMC1622834

[28] Hecht NB (1998) Molecular mechanisms of male germ cell differentiation. Bioessays 20: 555–561. 9723004 10.1002/(SICI)1521-1878(199807)20:7<555::AID-BIES6>3.0.CO;2-J

[29] Yamamoto M, Takahashi S, Onodera K, Muraosa Y, Engel JD (1997) Upstream and downstream of erythroid transcription factor GATA-1. Genes Cells 2: 107–115. 9167968 10.1046/j.1365-2443.1997.1080305.x

[30] Ketola I, Rahman N, Toppari J, Bielinska M, Porter-Tinge SB, et al (1999) Expression and regulation of transcription factors GATA-4 and GATA-6 in developing mouse testis. Endocrinology 140: 1470–1480. 10067876 10.1210/endo.140.3.6587

[31] Ketola I, Anttonen M, Vaskivuo T, Tapanainen JS, Toppari J, et al (2002) Developmental expression and spermatogenic stage specificity of transcription factors GATA-1 and GATA-4 and their cofactors FOG-1 and FOG-2 in the mouse testis. Eur.J.Endocrinol. 147: 397–406. 10.1530/eje.0.1470397 12213678

[32] Yomogida K, Ohtani H, Harigae H, Ito E, Nishimune Y, et al (1994) Developmental stage- and spermatogenic cycle-specific expression of transcription factor GATA-1 in mouse Sertoli cells. Development 120: 1759–1766. 7924983 10.1242/dev.120.7.1759

[33] Russell LB (1990) Patterns of mutational sensitivity to chemicals in poststem-cell stages of mouse spermatogenesis. Prog.Clin.Biol.Res. 340C: 101–113. 2199974

[34] Russell LD, Clermont Y (1977) Degeneration of germ cells in normal, hypophysectomized and hormone treated hypophysectomized rats. Anat.Rec. 187: 347–366. 10.1002/ar.1091870307 851237

[35] Tan KA, De GK, Atanassova N, Walker M, Sharpe RM, et al (2005) The role of androgens in sertoli cell proliferation and functional maturation: studies in mice with total or Sertoli cell-selective ablation of the androgen receptor. Endocrinology 146: 2674–2683. 15761038 10.1210/en.2004-1630

[36] Lin RJ, Nagy L, Inoue S, Shao W, Miller WH, et al (1998) Role of the histone deacetylase complex in acute promyelocytic leukaemia. Nature 391: 811–814. 9486654 10.1038/35895

[37] Wong CW, Privalsky ML (1998) Transcriptional repression by the SMRT-mSin3 corepressor: multiple interactions, multiple mechanisms, and a potential role for TFIIB. Mol.Cell Biol. 18: 5500–5510. 10.1128/mcb.18.9.5500 PMC109135 9710634

[38] Omori M, Yamashita M, Inami M, Ukai-Tadenuma M, Kimura M, et al (2003) CD8 T cell-specific downregulation of histone hyperacetylation and gene activation of the IL-4 gene locus by ROG, repressor of GATA. Immunity. 19: 281–294. 10.1016/s1074-7613(03)00210-3 12932361

[39] Cumming RC, Lightfoot J, Beard K, Youssoufian H, O’Brien PJ, et al (2001) Fanconi anemia group C protein prevents apoptosis in hematopoietic cells through redox regulation of GSTP1. Nat.Med. 7: 814–820. 10.1038/89937 11433346

[40] Niedzwiedz W, Mosedale G, Johnson M, Ong CY, Pace P, et al (2004) The Fanconi anaemia gene FANCC promotes homologous recombination and error-prone DNA repair. Mol.Cell 15: 607–620. 15327776 10.1016/j.molcel.2004.08.009

[41] Griswold MD (1998) The central role of Sertoli cells in spermatogenesis. Semin.Cell Dev.Biol. 9: 411–416. 10.1006/scdb.1998.0203 9813187

[42] Verhoeven G (1992) Local control systems within the testis. Baillieres Clin.Endocrinol.Metab 6: 313–333. 1616447 10.1016/s0950-351x(05)80152-1

[43] Mruk DD, Cheng CY (2004) Sertoli-Sertoli and Sertoli-germ cell interactions and their significance in germ cell movement in the seminiferous epithelium during spermatogenesis. Endocr.Rev. 25: 747–806. 10.1210/er.2003-0022 15466940

[44] De GK, Swinnen JV, Saunders PT, Schoonjans L, Dewerchin M, et al (2004) A Sertoli cell-selective knockout of the androgen receptor causes spermatogenic arrest in meiosis. Proc.Natl.Acad.Sci.U.S.A 101: 1327–1332. 14745012 10.1073/pnas.0308114100 PMC337052

[45] Djakiew D, Dym M (1988) Pachytene spermatocyte proteins influence Sertoli cell function. Biol.Reprod. 39: 1193–1205. 10.1095/biolreprod39.5.1193 3219390

[46] Onoda M, Djakiew D (1991) Pachytene spermatocyte protein(s) stimulate Sertoli cells grown in bicameral chambers: dose-dependent secretion of ceruloplasmin, sulfated glycoprotein-1, sulfated glycoprotein-2, and transferrin. In Vitro Cell Dev.Biol. 27A: 215–222. 10.1007/BF02630919 1903382

[47] Syed V, Gomez E, Hecht NB (1999) Messenger ribonucleic acids encoding a serotonin receptor and a novel gene are induced in Sertoli cells by a secreted factor(s) from male rat meiotic germ cells. Endocrinology 140: 5754–5760. 10579341 10.1210/endo.140.12.7194

[48] Holdcraft RW, Braun RE (2004) Androgen receptor function is required in Sertoli cells for the terminal differentiation of haploid spermatids. Development 131: 459–467. 14701682 10.1242/dev.00957

[49] LaVoie HA (2003) The role of GATA in mammalian reproduction. Exp.Biol.Med.(Maywood.) 228: 1282–1290. 14681544 10.1177/153537020322801107

[50] Viger RS, Taniguchi H, Robert NM, Tremblay JJ (2004) Role of the GATA family of transcription factors in andrology. J.Androl 25: 441–452. 15223831 10.1002/j.1939-4640.2004.tb02813.x

[51] Tsuzuki S, Enver T (2002) Interactions of GATA-2 with the promyelocytic leukemia zinc finger (PLZF) protein, its homologue FAZF, and the t(11;17)-generated PLZF-retinoic acid receptor alpha oncoprotein. Blood 99: 3404–3410. 11964310 10.1182/blood.v99.9.3404

[52] Merika M, Orkin SH (1993) DNA-binding specificity of GATA family transcription factors. Mol.Cell Biol. 13: 3999–4010. 10.1128/mcb.13.7.3999-4010.1993 PMC359949 8321207

[53] Watamoto K, Towatari M, Ozawa Y, Miyata Y, Okamoto M, et al (2003) Altered interaction of HDAC5 with GATA-1 during MEL cell differentiation. Oncogene 22: 9176–9184. 14668799 10.1038/sj.onc.1206902

[54] Gaughan L, Logan IR, Coo S, Neal DE, Robson CN (2002) Tip60 and histone deacetylase 1 regulate androgen receptor activity through changes to the acetylation status of the receptor. J.Biol.Chem. 277: 25904–25913. 10.1074/jbc.M203423200 11994312

[55] Qamar I, Park E, Gong EY, Lee HJ, Lee K (2009) ARR19 (androgen receptor corepressor of 19 kDa), an antisteroidogenic factor, is regulated by GATA-1 in testicular Leydig cells. J.Biol.Chem. 284: 18021–18032. 10.1074/jbc.M900896200 PMC2709340 19398553

[56] Jeong BC, Hong CY, Chattopadhyay S, Park JH, Gong EY, et al (2004) Androgen receptor corepressor-19 kDa (ARR19), a leucine-rich protein that represses the transcriptional activity of androgen receptor through recruitment of histone deacetylase. Mol.Endocrinol. 18: 13–25. 10.1210/me.2003-0065 14576337

[57] Tang CJ, Lin CY, Tang TK (2006) Dynamic localization and functional implications of Aurora-C kinase during male mouse meiosis. Dev.Biol. 290: 398–410. 10.1016/j.ydbio.2005.11.036 16386730

[58] Shu SK, Liu Q, Coppola D, Cheng JQ (2010) Phosphorylation and activation of androgen receptor by Aurora-A. J.Biol.Chem. 285: 33045–33053. 10.1074/jbc.M110.121129 PMC2963369 20713353

[59] Khan J, Ezan F, Cremet JY, Fautrel A, Gilot D, et al (2011) Overexpression of active Aurora-C kinase results in cell transformation and tumour formation. PLoS.One. 6: e26512. 10.1371/journal.pone.0026512 PMC3203144 22046298

